# Association of Stillbirths with Maternal and Fetal Risk Factors in a Tertiary Care Hospital in South India

**DOI:** 10.1155/2021/8033248

**Published:** 2021-07-22

**Authors:** Rajshekher V. Mali, Anita Dalal, Romana Khursheed, Aditya Gan

**Affiliations:** Department of Obstetrics and Gynecology, Jawaharlal Nehru Medical College, KLE Academy of Higher Education and Research (Deemed to Be University), Belagavi-590010, Karnataka, India

## Abstract

**Background:**

Birth of a fetus with no signs of life after a predefined age of viability is a nightmare for the obstetrician. Stillbirth is a sensitive indicator of maternal care during the antepartum and intrapartum period. Though there has been a renewed global focus on stillbirth as a public health concern, the decline in stillbirth rate (SBR) has not been satisfactory across the nations, with a large number of stillbirths occurring in the low- to middle-income countries (LMICs). Hence, the study was carried out to analyze maternal and fetal risk factors and their association with stillbirths in a tertiary care center in South India.

**Methods:**

This observational prospective study included pregnant women with stillbirth beyond 20 weeks of gestation or fetal weight more than 500 grams. Stillbirths were classified according to the simplified causes of death and associated conditions (CODAC) classification. Association between the risk factor and stillbirths was calculated with chi-square test and odds ratio with 95% confidence interval.

**Results:**

There were 171 stillbirths (2.97%) among total 5755 births. The SBR was 29.71/1000 births. Risk factors such as preterm delivery (OR: 22.33, 95% CI: 15.35–32.50), anemia (OR: 21.87, 95% CI: 15.69–30.48), congenital malformation (OR: 11.24, 95% CI: 6.99–18.06), abruption (OR: 10.14, 95% CI: 6.43–15.97), oligohydramnios (OR: 4.88, 95% CI: 3.23–7.39), and hypertensive disorder (OR: 3.01, 95% CI: 2.03–4.46) were significantly associated with stillbirths. The proportion of intrapartum stillbirths was found to be 5 (3%) among the study population.

**Conclusion:**

Highest prevalent risk factors associated with stillbirth are anemia and prematurity. Intrapartum stillbirths can be reduced significantly through evidence-based clinical interventions and practices in resource-poor settings. There is a need to provide and assure access to specialized quality antenatal care to pregnant women to control the risk factors associated with stillbirths.

## 1. Background

Stillbirth is defined generally as delivery of a fetus, following a predefined period of gestation and before complete expulsion from its mother, showing no signs of life and who cannot be resuscitated following birth [1]. In India, a fetus ≥20 weeks of gestation with no signs of life is considered stillborn [2]. Stillbirth is the most prevalent adverse outcome of pregnancy. Death of a viable fetus is one of the most distressing events for the parents as well as for the obstetrician. Also, it is a sensitive marker of quality and equity of healthcare. The stillbirth rate (SBR) was 18.4/1000 total births worldwide in 2015 [3]. The low- to middle-income countries (LMICs) contribute to 98% of stillbirths, and yearly, 592,100 stillbirths occur in India, with a rate of 22/1000 total births (World Health Organization (WHO)) [4, 5]. The Every Newborn Action Plan (ENAP) in year 2014 has proposed a stillbirth rate target of 12 or fewer per 1000 births by year 2030 [6]. The 2019 ENAP progress report has depicted that 59% of the reporting countries will fail to achieve the stillbirth interim target of 14/1000 or less by 2020 [6]. The annual reduction in stillbirths across the globe is 2%, which is lesser than the decline in maternal mortality (3%) and under-five child mortality (4.5%) [7]. Though stillbirths comprise a large proportion of preventable deaths, it is challenging to determine their cause as the fetus is not directly observed when death occurs and the events prior to it. Furthermore, there is lag in knowledge in understanding the conditions and contexts before stillbirth occurs. The 2019 ENAP progress report recommends to intensify research into causes of stillbirth so that the results of these studies direct policy developments to reach the global target of stillbirth reduction.

Assigning a cause to the stillbirth and establishing its association with different maternal and fetal factors can help to prioritize interventions to improve birth outcomes in resource-poor areas. The present study attempted to identify the possible risk factors and their association with stillbirths in a tertiary care center in South India.

## 2. Materials and Methods

### 2.1. Study Setting

The present prospective, observational study was conducted from January 1, 2017, to December 31, 2017, at Karnataka Lingayat Education Academy of Higher Education and Research's Dr. Prabhakar Kore Charitable Hospital, attached to Jawaharlal Nehru Medical College, Belagavi, South India. It is a 1200-bed multispecialty hospital which is a referral center for the neighboring states. The labor ward is having total 64 beds and two operation theatres. Among these, 20 beds are obstetric intensive care beds. Annually, there are approximately 6000 deliveries. The labor ward is managed round the clock by a team of consultants, nurses, and postgraduates in obstetrics and gynecology (OBG), pediatrics, and anesthesiology departments. The institutional evidence-based protocols are used as guidelines for the management of high-risk cases.

### 2.2. Study Participants

#### 2.2.1. Inclusion Criteria

All the women who were admitted to the labor ward and had stillbirth and consented for enrollment were included in the study. Written informed consent was obtained from all the study participants.

#### 2.2.2. Exclusion Criteria

The exclusion criterion was gravidas having live births.

For the present work, ethical approval was obtained from the Institutional Ethics Committee of Jawaharlal Nehru Medical College of KLE Academy of Higher Education and Research, Belagavi, which certified that the study was performed in accordance with the ethical standards as laid down in the 1964 Declaration of Helsinki and its later amendments or comparable ethical standards.

### 2.3. Data Collection Procedure

The study proforma was discussed and verified by a group of obstetricians in a departmental review meeting. The pretesting of the study proforma was done before starting the data collection, and necessary changes were made and confirmed. The stillbirth data were recorded by a postgraduate medical student in the Obstetrics and Gynecology department, who was trained to get acquainted with the methodology of the research study. They were verified by an assigned professor in OBG.

The study participants were analyzed in terms of age, gravidity, and socioeconomic status (as per the 2016 Kuppuswamy scale for the urban population and the 2016 BG Prasad scale for the rural population) [8]. In the index pregnancy, details of antenatal checkups, medical illnesses, presence of obstetric complications, and other significant illnesses were evaluated. The gestational age was assigned as per the first trimester dating ultrasound or by Sonocare software if the dating scan was not available. Complete general physical examination/systemic examination and obstetric examination were conducted. Absence of fetal heart sound was confirmed by ultrasound examination. Mode of delivery and birth weights of fetuses and presence or absence of malformation were noted.

Every stillbirth was discussed in detail in perinatal death audit in the department of OBG of the institution, and cause of death was assigned based on history, clinical examination, and available investigations by a multidisciplinary team, consisting of a senior obstetrician and neonatologist. The cause of death was further classified as per the simplified CODAC, system of classification for stillbirths [9].

### 2.4. Operational Definitions

- Registered gravida: a woman who had 4 or more antenatal visits
- Preterm stillbirth: stillbirth occurring before 37 completed weeks of gestation
- Postterm stillbirth: stillbirth occurring after 42 weeks of gestation
- Antepartum stillbirth: the intrauterine fetal demise occurred before the onset of labor
- Intrapartum stillbirth: the intrauterine fetal demise occurred during labor
- Early stillbirth: stillbirths which occurred between 20 and 27 weeks and 6 days
- Late stillbirth: stillbirths which occurred after 28 weeks
- Stillbirth: it was defined as birth of a baby, with no signs of life occurring after 20 weeks of pregnancy or with >500 grams of fetal weight [9]
- Extreme prematurity: birth before 28 weeks of gestation

## 3. Results

### 3.1. The Stillbirth Rate (SBR)

A total of 171 stillbirths (2.97%), with the stillbirth rate of 29.71/1000 births among 5755 total births, were observed.

### 3.2. The Sociodemographic and Clinical Presentation of the Participants

According to our results, 88 (51.46%) and 83 (48.54%) stillbirths were observed among the rural and urban population, respectively. The majority of stillbirths, 51 (61.45%) and 36 (40.91%) in the urban and rural population, respectively, belonged to class 3 (lower middle class in the Kuppuswamy scale and middle class in the modified BG Prasad scale). Among registered gravidas, 77 stillbirths (45%) were noted, and 94 stillbirths (55%) were noted in unregistered gravidas. Most of the stillbirths (*n* = 140) (81.9%) were found in the women of 20–30 years age group. Stillbirths were more common in primigravida that is 78 stillbirths (45.61%) followed by 43 stillbirths (25.15%) in gravida-2, 32 stillbirths (18.71%) in gravida-3, and 18 stillbirths (10.53%) in gravida-4 and above. It was observed that 136 stillbirths (79.53%) were delivered vaginally, and 35 stillbirths (20.47%) were delivered through cesarean section (18 abruption, 6 eclampsia with end-organ damage, 5 previous LSCS, 3 fetal distress, 1 placenta previa, 1 rupture uterus, and 1 obstructed labor) (Table 1).

### 3.3. Stillbirths at Different Gestational Ages

**Figure 1:**
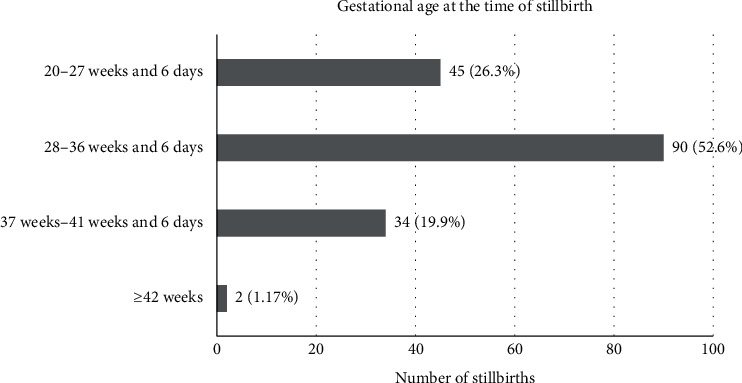
Number of stillbirths at different gestational ages.

According to Figure 1, a total of 45 stillbirths (26.32%) occurred in women with the gestational age of 20–27 weeks and 6 days, 90 stillbirths (52.63%) were noted at gestational age between 28 weeks and 36 weeks and 6 days period of gestation, 34 stillbirths (19.88%) were seen at gestational age between 37 and 41 weeks and 6 days, and 2 stillbirths (1.17%) occurred at gestational age more than 42 weeks. The total number of preterm (<37 weeks) stillbirths was 135 (78.94%), followed by 34 (19.88%) in term (37–41 weeks) and 2 (1.17%) in postterm (≥42 weeks) gestation (Table 1).

### 3.4. Stillbirths in Antepartum and Intrapartum Period

**Figure 2:**
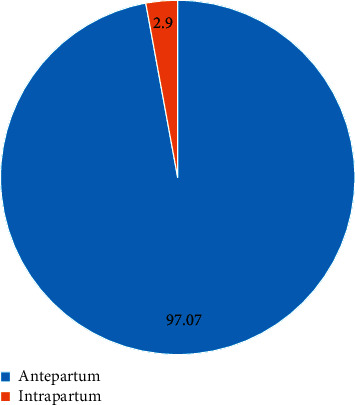
Proportion of stillbirths in the antepartum and intrapartum period.

The proportion of antepartum and intrapartum stillbirths is shown in Figure 2. There were 5 stillbirths (2.9%) in intrapartum period and 166 stillbirths (97.07%) in antepartum period.

### 3.5. The Distribution of Birth Weight among Stillbirths

**Figure 3:**
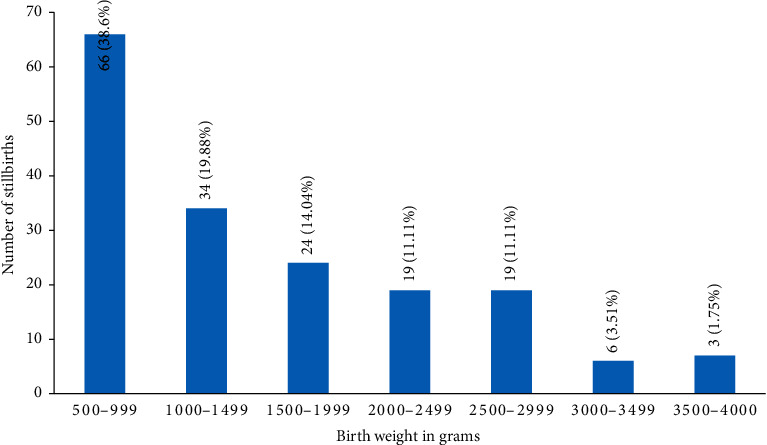
Birth weight (in grams) distribution in stillbirths.

It was observed to be as follows: the maximum number of stillbirths, that is, 66 (38.59%), had birth weight between 500 and 999 grams, followed by 34 stillbirths (19.88%) with birth weight between 1000 and 1499 grams.19 stillbirths (11.11%) were observed in both 2000–2499 grams and 2500–2999 grams categories. Six stillbirths (3.51%) were reported between 3000 and 3499 grams, and 3 stillbirths (1.75%) were present between 3500 and 4000 grams birth weight (Figure 3).

### 3.6. Causes of Death and Associated Condition (CODAC)

The common causes of death in the order of decreasing frequency were fetal causes (50 (29.24%)), maternal conditions (42 (24.56%)), placental causes (28 (16.37%)), and congenital malformations (25 (14.62%)), whereas unknown causes were 16 (9.36%). Extreme prematurity, hypertensive disorders in pregnancy, abruption, and congenital malformations were present in 45 (26.32%), 33 (19.3%), 27 (15.79%), and 25 (14.62%), respectively (Table 2).

### 3.7. Association of Stillbirths with Maternal and Fetal Risk Factors

Table 3 describes that preterm delivery, hypertensive disorders, anemia, abruption, congenital malformations, and oligohydramnios were significantly associated with stillbirths (*p* < 0.05). Through unadjusted odds ratio, it was noted that odds of stillbirths were 22.33 (95% CI: 15.35–32.50) times higher for the stillbirths with preterm delivery than the stillbirths with full-term delivery. An odd of stillbirths for the subjects with congenital anomalies was 11.24 (95% CI: 6.99–18.06) times higher than the subjects without congenital anomalies. Also, it was observed that the odds of stillbirths were 3.01 (95% CI: 2.03–4.46) times higher for the subjects with hypertensive disorder than subjects without hypertensive disorder. The odds of stillbirth were 10.14 (95% CI: 6.43–15.97), 4.88 (95% CI: 3.23–7.39), and 21.87 (95% CI: 15.69–30.48) times higher for the subjects with abruption, oligohydramnios, and anemia than the subjects without abruption, oligohydramnios, and anemia, respectively.

## 4. Discussion

Among 5755 total births, 171 stillbirths (2.97%) were observed. The SBR was calculated to be 29.71/1000 births. According to a report in year 2018, the national range of SBR in India is 22 to 66/1000 births [10]. It has decreased from 31.3/1000 births in year 2010 to 23.8/1000 births in year 2016 [11]. Given the current rate of annual decline in Indian stillbirths of 4.5%, a higher diminution of 5.8%, in the current rates, is vital to achieve the ENAP goal [11].

The stillbirths were more in unregistered women as compared to registered women, that is, 94 (54.97%) and 77 (45.03%) stillbirths, respectively, in the study setup. This is in concurrence to a study conducted by Rajagopal et al. (unregistered stillbirths 54.4% vs. registered stillbirths 45.5%) [12]. The diagnosis and surveillance of high-risk pregnancies by skilled health personnel and prompt effective management of complications in registered gravidas can explain the low stillbirths in the registered women.

A majority of the stillbirths, that is 88 (51.46%), were found in women from the rural population. Dandona et al. also found higher stillbirths of 62.4% in the rural population [13]. In India, though there is implementation of financial incentive programs for pregnant women attending health services, the quality of care in peripheral health facilities is usually compromised, and such women are referred late to tertiary care centers. It is observed that 20–30% of stillbirths result from suboptimal obstetric care [14]. The higher prevalence of stillbirths in the rural population suggests the need for improved obstetric care as well as availability of emergency services in the rural settings. The study revealed that most of the pregnant women who had stillbirth were from lower middle and middle socioeconomic status, 51 (61.45%) and 36 (40.91%), respectively. The socioeconomic status influences the pregnancy outcome and determines health-seeking behavior of the women in accessing antenatal, intranatal, and emergency obstetric care. The comparable higher stillbirths in lower and middle socioeconomic groups were noted in a study by Asalkar et al. (43, 33) [15].

Though advanced maternal age is a known risk factor for both increased perinatal morbidity and mortality, the majority of the stillbirths (140 (82%)) were seen in the women between the age group of 20 and 30 years, similar higher rates were seen in a study conducted by Rajagopal et al. (71.4%) [12]. In India, a higher prevalence of early marriage and completion of family before 35 years of life in women can explain the higher number of births and stillbirths in this age group. Lack of awareness about pregnancy-related complications and poor access to obstetric care facilities add fuel to this problem.

It was observed that stillbirths were more common in the primigravida (78 (45.61%)) among the study population. The relationship between gravidity and prevalence of the stillbirth is comparable to previous studies conducted by Asalkar et al. (primigravida 48.3%) and Prasanna et al. (primigravida 49.8%) [15, 16]. A study conducted by Saleem et al. also reported an increased risk of stillbirth in the first and after the fifth pregnancy [11]. In this study, almost half of stillbirths (90 (52.63%)) were late stillbirths (28 weeks–36 weeks and 6 days), whereas one-fourth of stillbirths (45 (26.31%)) were early stillbirths (20–27 weeks and 6 days).

The proportion of preterm stillbirths was 135 (78.94%), and it was highest among stillbirths at different gestational ages in the study population. The higher number of late stillbirths is comparable to other studies such as Agbata et al. (81%), Devi et al. (57%), and Rajagopal et al. (75%) [10, 12, 17]. The prevalence of low birth weight (<2.5 kg) including very low birth weight (1000–1499 grams) and extremely low birth weight (500–999 grams) was 143 (83.62%) collectively in the study population. Similar results were seen by Sharma et al. (78.8%) [9]. Fetal growth restriction and prematurity are important causes of low-birth-weight stillbirths.

In this study, intrapartum deaths were 5 (3%), and antepartum deaths were 166 (97%). Lawn et al. estimated that intrapartum stillbirth rate was about 39% in middle-income countries [7]. Intrapartum stillbirths are quality indicators of a health institute. Low intrapartum stillbirths in this study reflected standard institutional protocol-based intrapartum care, close monitoring of the pregnant women during labor, availability of operation theatres and skilled personnel round the clock, and conduct of perinatal death audit for every stillbirth counted in the tertiary care center. Goldenberg et al. declared that, with every percentage rise in CS rate from 0 to 8%, intrapartum stillbirths drop by 1.6/1000 births in LMIC [18].

It was found that cesarean sections were 35 (20.47%) and vaginal deliveries were 136 (79.53%) among stillbirths in this study. As the numbers of antepartum stillbirths were high in this study, such stillbirths are induced and delivered by the vaginal route unless there is a contraindication. For reducing antepartum stillbirths, there is a need to ensure access to quality care in terms of comprehensive interventions focusing on social, nutritional, and healthcare needs in developing nations as complications during the antepartum period are often associated with poor outcome of pregnancy. The causes of death were categorized as per the simplified CODAC system of classification among the study population. The common causes of death in the order of decreasing frequency were fetal causes (50 (29.24%)), maternal conditions (42 (24.56%)), placental causes (28 (16.37%)), and congenital malformations (25 (14.62%)), whereas unknown causes were 16 (9.36%). In a study conducted by Sharma et al., maternal conditions were 39.12% and unknown causes were 19.87% and ranked the highest in the causes of stillbirths in a tertiary care center in North India [9].

The prevalence of preterm delivery, hypertensive disorders of pregnancy, abruption, and congenital malformations with stillbirths was estimated to be 135 (78.94%), 33 (19.29%), 27 (15.79%), and 25 (14.62%) in the present study, and they were statistically significant (*p* < 0.05), with odds of stillbirth 22.33 (95% CI: 15.35–32.50), 3.01 (95% CI: 2.03–4.46), 10.14 (95% CI: 6.43–15.97), and 11.24 (95% CI: 6.99–18.06) times higher, respectively. Neogi et al. declared that the possibility of mothers with preterm delivery was 4.5 times higher of having stillbirth compared to full-term delivery [19]. The preterm stillbirths in this study include both spontaneous and induced preterm for different medical and obstetric complications. Significant association of the stillbirth with maternal hypertension was also noticed in many studies conducted in India and other countries [4, 20–22]. As abruption is commonly associated with hypertensive disorders of pregnancy, there is a need to follow stringent monitoring of women with preeclampsia, and timely intervention is crucial to reduce the burden of stillbirths associated with hypertensive disorders of pregnancy. Antepartum hemorrhage was reported as one of the top five causes of stillbirths in all income setting countries (low, middle, and high) [23]. Anemia and oligohydramnios were seen in 77 (45.03%) and 30 (17.54%) stillbirths in the study population with a statistically significant association (*p* < 0.0001) and higher odds of 21.87 and 4.88 compared to women without them. Altijani et al. also observed 35% higher odds of stillbirths with anemia compared with women who did not have anemia [4]. A higher odd for oligohydramnios was also seen in a study conducted by Zile et al. [24]. Congenital malformations were present in 25 stillbirths (14.62%) (*p*=0.0005) and had 11.24 times higher odds of stillbirth in the present study. It was noted that most of the lethal anomalies were detected late in the pregnancy. Thus, timely anomaly scans at or before 20 weeks of gestation are highly recommended to detect lethal congenital anomalies early in pregnancy so that these are terminated within legal limits, and burden of stillbirths due to congenital anomalies is reduced.

Though fetal growth restriction, fetal distress, and diabetes mellitus in pregnancy are potential risk factors for stillbirths, they were not significantly associated with stillbirths in this study. Good intranatal care and institutional protocol-based management of high-risk pregnancy could be a reason for comparatively low stillbirths in these groups. Universal screening of pregnant women for diabetes and multidisciplinary management results in better compliance and reduced complications in diabetic pregnancy. We follow routine growth scan for all the antenatal females in the third trimester. This ensures early pick up of fetal growth restriction and low-birth-weight fetuses who further undergo rigorous follow-up with Doppler studies and need-based early termination.

### 4.1. Strengths and Limitations of the Study

Our study data are the first (to the best of our knowledge) to report the potential of reducing intrapartum stillbirths in a LMIC setup, with standard protocol-based institutional management of high-risk pregnancies. The real challenge in reducing overall stillbirths is to decrease the antepartum stillbirths. Since autopsies or other tests were not performed on stillborn babies, it is difficult to establish any causation in the unknown category. There are limitations in performing fetal autopsy in stillbirths. Unwillingness by the parents due to financial and social concerns was identified as the most prevalent factor. Being a LMIC setup and due to financial constraints, certain advanced tests such as polymerase chain reaction (PCR) for infective pathology and cytogenetic analysis and MITS (minimally invasive tissue sampling) could not be performed. Future studies should focus on investigating their role in assessing the cause of death in stillbirths.

## 5. Conclusion

Preterm labor, anemia, congenital malformations, abruption, hypertensive disorder of pregnancy, and oligohydramnios were positively associated with stillbirths in this study. All the risk factors can be minimized by screening for early detection and prompt effective timely intervention in resource-poor settings. Perinatal death audit which includes thorough revision of stillbirths is a promising and practical quality improvement method that can be implemented in different settings and has potential to reduce the perinatal deaths. There is a need to regularize uniform protocols for antenatal and intranatal care in both urban and rural settings for a better neonatal outcome.

## Figures and Tables

**Table 1:** Sociodemographic and clinical characteristics of gravidas and their stillborn in Dr. Prabhakar Kore Charitable Hospital (January 1, 2017–December 31, 2017).

Variable		Subvariable	Number	Percentage
Residence		Urban	83	48.54
		Rural	88	51.46

Socioeconomic status	Urban population (Kuppuswamy scale) (*n* = 83)^*∗*^	I	1	1.2
		II	14	16.87
		III	51	61.45
		IV	13	15.66
		V	4	4.82
	Rural population (BG Prasad scale) (*n* = 88)^#^	*R*1	0	0
		*R*2	22	25
		*R*3	36	40.91
		*R*4	26	29.55
		*R*5	4	4.55

Antenatal care		Registered	77	45.03
		Unregistered	94	54.97

Age distribution of women with stillbirths		<20 years	17	9.97
		>20–<30 years	140	81.9
		>30 years	14	8.19

Stillbirths in different gravidity		Gravida-1	78	45.61
		Gravida-2	43	25.15
		Gravida-3	32	18.71
		≥Gravida-4	18	10.53

Period of gestation at the time of stillbirth		Preterm (<37 wk)	135	78.94
		Term (37–41 wk)	34	19.88
		Postterm (>42 wk)	2	1.16

Mode of delivery in stillbirths		Vaginal delivery	136	79.53
		Cesarean section	35	20.47

^∗^ and ^#^indicate the percentage taken among the total number of urban and rural populations, respectively, Kuppusamy scale—I: upper class; II: upper middle class; III: lower middle class; IV: upper lower class; V: lower class; BG Prasad scale—*R*1: upper class; *R*2: upper middle class; *R*3: middle class; *R*4: lower middle class; *R*5: lower class.

**Table 2:** Causes of stillbirths as per simplified CODAC in Dr. Prabhakar Kore Hospital (January 1, 2017–December 31, 2017).

Cause of death	Subvariable	Number of stillbirths	Total stillbirths (%)
Infections (*n* = 2) (1.16%)	Chorioamnionitis	1	0.58
	Toxoplasmosis	1	0.58

Intrapartum (*n* = 5) (2.92%)	Fatal distress	3	1.75
	Prolonged labor/obstructed labor	1	0.58
	Rupture uterus	1	0.58

Congenital anomaly (*n* = 25) (14.62%)			
Other fetal causes (*n* = 50) (29.24%)	Extreme prematurity	45	26.32
	Hydrops of unknown origin	5	2.92

Cord accidents (*n* = 3) (1.75%)	Loops	2	1.17
	Cord prolapse	1	0.58

Placental causes (*n* = 28) (16.37%)	Abruptio placenta	27	15.79
	Placenta previa	1	0.58

Maternal conditions (*n* = 42) (24.56%)	Hypertensive disorder	33	19.3
	Preeclampsia	26	15.2
	Eclampsia	6	3.51
	Chronic hypertension	1	0.58
	Diabetes	7	4.09

Unknown		16	9.36
Total	171	100	
Associated perinatal	Small for gestational age	30	17.54
	Multiple pregnancies	2	1.17

Associated maternal	Anemia	77	45.03
	Oligohydramnios	30	17.54

**Table 3:** Association of stillbirths with different maternal and fetal factors.

Maternal and fetal risk factors	Number of women with risk factors among the total number of births (*n *=* *5755)	Number of stillbirths with risk factors	Percentage	Odds ratio (95% CI)	*p* value
Hypertensive disorders of pregnancy	454	33	19.29	3.01 [2.03–4.46]	<0.0001^*∗*^
Abruption	131	27	15.79	10.14 [6.43–15.97]	0.0005^*∗*MC^
Oligohydramnios	269	30	17.54	4.88 [3.23–7.39]	<0.0001^*∗*^
Anemia	282	77	45.03	21.87 [15.69–30.48]	<0.0001^*∗*^
Preterm delivery	943	135	78.94	22.33 [15.35–32.50]	<0.0001^*∗*^
Premature rupture of membranes	682	15	8.77	0.73 [0.43–1.25]	0.2506
FGR	915	30	17.54	1.16 [0.78–1.74]	0.458
Fetal distress	70	03	1.75	1.52 [0.47–4.87]	0.7271^MC^
DM	372	7	4.09	0.63 [0.29–1.35]	0.2309
Congenital malformations	111	25	14.62	11.24 [6.99–18.06]	0.0005^*∗*MC^
Maternal infection	72	2	1.06	0.96 [0.23–3.95]	>0.99^MC^

MC: Monte Carlo's simulation used in chi-square test; ^∗^indicates significance; FGR: fetal growth restriction; DM: diabetes mellitus.

## Data Availability

Preliminary data are available on request from the corresponding author. Data related to confidentiality of the subject are restricted.

## Abbreviations

CODAC: Cause of death and associated condition
ENAP: Every Newborn Action Plan
SBR: Stillbirth rate
WHO: World Health Organization
NICU: Neonatal intensive care unit
PCR: Polymerase chain reaction.
